# A. P. Southwick, D.D.S.

**Published:** 1899-07

**Authors:** 


### A. P. SOUTHWICK, D.D.S.

THE intelligence of the sudden and unexpected death of our friend and colaborer, Dr. A. P. Southwick, of Buffalo, N. Y., awakened within our hearts emotions of the most profound sorrow, while bowing to the Divine Wisdom which has taken him from us. We improve this opportunity of paying a grateful tribute to his memory, and to give expression to our appreciation of him as a man and coworker in the dental profession.

All will bear testimony that he was a genial and cordial gentleman of noble impulses, full of kindly charities, and always awake to the interests and progress of his calling. He was enterprising in all his undertakings. The State Board of Dental Examiners, of which he was so long its president, the State Society, which likewise enjoyed his wisdom in council and labor for many years, as well as the Dental Department of the University of Buffalo, which has lost an earnest worker and most efficient teacher, will deplore their loss.

